# Advancing Geriatric Dental Education: A Scoping Review of Barriers and Facilitators in Undergraduate Curricula

**DOI:** 10.3390/dj14090548

**Published:** 2026-09-01

**Authors:** Alice Kit Ying Chan, Lindsey Lingxi Hu, Joanna Cheuk Yan Hui, Chun Hung Chu

**Affiliations:** Faculty of Dentistry, The University of Hong Kong, Hong Kong 999077, China; dralice@hku.hk (A.K.Y.C.); hu_lingxi@connect.hku.hk (L.L.H.); cyjaonna@hku.hk (J.C.Y.H.)

**Keywords:** older adults, elderly, dental education, geriatric dentistry, oral health, prevention

## Abstract

**Background:** Geriatric dentistry prepares dentists to care for an aging population. Yet, its integration into undergraduate curricula remains inconsistent. **Objectives:** To map evidence on the barriers to and facilitators of the implementation and development of undergraduate geriatric dental education. **Methods:** A scoping review was conducted using systematic searches of PubMed, Scopus, and Embase for English-language original research articles published from 1 January 2016 onward. Two reviewers screened titles, abstracts, and full texts independently against eligibility criteria. Studies were included if they examined barriers and/or facilitators related to undergraduate geriatric dental education. Data were extracted independently and synthesized descriptively to identify themes and evidence gaps. No critical appraisal was performed. **Results:** This review identified 1376 studies and included 11 studies, including seven cross-sectional and four cohort studies. Barriers clustered into three themes: structural and workforce-related barriers (resource constraints and a lack of equipped/trained staff), curricular and regulatory barriers (overcrowded curricula and a lack of curriculum guidelines), and interprofessional and attitudinal barriers (limited interprofessional collaboration and negative attitudes among students and teaching staff towards older adults). Facilitators were grouped into three themes: pedagogical and experiential learning strategies (didactic teaching, clinical training, extramural exposure, simulation-based training, and communication training), curricular and policy enablers (integrated curricula and national guidelines), and institutional and faculty support (staff support). **Conclusions:** This review identifies the multifaceted, modifiable educational, institutional, and policy factors that influence the implementation and development of geriatric dental education. By consolidating the existing evidence, this review provides a foundation for curriculum development and policy planning to prepare graduates to deliver age-responsive oral healthcare.

## 1. Introduction

The global population is aging rapidly, with projections indicating that by 2050, one in six individuals will be an older adult aged 65 or above [1]. Advances in preventive measures and increases in dental awareness enable older people to retain more teeth throughout their lifetime. However, older adults remain susceptible to oral diseases such as root caries and periodontal disease, owing to age-related systemic, cognitive and physical changes [2]. Multimorbidity and polypharmacy can negatively affect salivary gland function, thereby increasing the risk of dental caries. Additionally, medical conditions like diabetes and cardiovascular diseases are associated with periodontal disease [3]. Cognitive and physical decline can impede their daily oral hygiene practices, which are essential for maintaining oral health, and hinder their access to dental care [4]. As the older population grows, the burden of oral diseases in this age group will reach unprecedented levels for individuals, society and healthcare systems [2].

Providing oral care to older adults presents unique challenges for dental professionals. Management approaches suitable for other adult groups may not be appropriate or sufficient for older patients. Age-related declines in systemic, cognitive and physical health complicate routine dental procedures like scaling or restorations, often necessitating additional or specialized care. For example, older adults with dysphagia should be positioned upright during scaling to prevent inadvertent aspiration, which could lead to pneumonia. Monitoring vital signs, such as blood pressure, during dental treatment may also be necessary for medically compromised older patients [5,6]. Dentists willing to treat older adults should ensure their clinical settings are equipped with appropriate facilities and equipment. This includes reserving extra space for wheelchair access and, in some countries, installing wheelchair recliners to allow older patients with mobility limitations to receive dental treatment without requiring a transfer [7]. Furthermore, dental professionals should strengthen their listening and communication skills to effectively explain oral conditions and treatment plans, especially since many older patients may experience some degree of cognitive or hearing decline. Due to the additional skills, knowledge and costs involved, many dental professionals are reluctant to treat older patients [8].

A cross-sectional study found that 30% of dentists felt inadequately equipped with the knowledge and skills to manage older patients with complex medical conditions, and 40% considered their dental education insufficient in geriatric dentistry [8]. Another survey revealed that 80% of dentists held some negative attitudes rooted in ageism, which hindered their willingness to treat older people [9]. Since general dental practitioners are the primary providers of oral care for older adults, it is crucial that their undergraduate training include the proper attitudes, knowledge and clinical skills to meet the increasing oral health demands of this population. Evidence suggests that exposure to geriatric dentistry during undergraduate education can enhance dental students’ confidence and willingness to manage older patients after graduation [10]. Accordingly, the working group at the 2024 Asia Pacific Dental Congress recommended integrating geriatric dentistry into undergraduate curricula as a strategy to promote oral health among the aging population [11].

Although the importance of including geriatric dentistry in dental education has been recognized for decades, its implementation remains inconsistent among dental schools. High-income countries such as the United States, Japan and several European countries have incorporated geriatric dental education into most dental schools; however, not all provide clinical training in this field. Reports indicate that approximately one-third of European dental students and nearly half of U.S. dental students have no experience treating older patients before graduation [12]. In middle- and low-income countries, geriatric dental education is often still in its early stages. For instance, despite China’s rapidly growing older population, less than 5% of dental schools include geriatric dentistry in their undergraduate curricula. Similarly, Brazil, recognized as the first country to establish geriatric dentistry as a dental specialty, offers geriatric dental education in only about one-quarter of its dental schools [12]. The barriers to and facilitators of implementing geriatric dental education vary across countries; however, no recent scoping review has comprehensively synthesized the evidence on this topic. Therefore, the aim of this scoping review is to map evidence on the current barriers to and facilitators of the implementation and development of undergraduate geriatric dental education.

## 2. Methods

This scoping review was conducted and reported in accordance with the Preferred Reporting Items for Systematic Reviews and Meta-Analyses (PRISMA) Extension for Scoping Reviews (PRISMA-ScR) Checklist (Supplementary Materials: Table S1) [13]. The review protocol was registered on 6 May 2026 with the Open Science Framework (Registration DOI: 10.17605/OSF.IO/7FAHN).

The review aimed to address the following two questions:

1. What is known from the literature about the challenges of implementing geriatric dental education in undergraduate curricula over the past 10 years?
2. What factors or strategies have been identified in the literature as facilitators of enhancing geriatric dental education in undergraduate curricula?

### 2.1. Search Strategy

Two independent researchers (AKC and LH) conducted the literature search across three electronic databases: PubMed, Scopus and Embase. The search used the following keywords: (“geriatric dentistry” OR “older adults” OR aged OR elderly OR elders) AND (“dental education” OR “dental curriculum” OR “dental training” OR “dental teaching”) AND (challenges OR barriers OR difficulties OR obstacles OR address OR overcome OR strategies OR facilitators OR solutions). No gray literature search was conducted. The final search was completed on 31 May 2026. The full search strategy is detailed in the Supplementary Materials: Document S1.

### 2.2. Search Selection and Eligibility Criteria

After removing duplicates, the two researchers independently screened and selected the titles and abstracts of the retrieved literature based on the predefined inclusion and exclusion criteria outlined in Table 1, using the population, concept and context framework [14].

Given the focus of this review on current barriers to and facilitators of implementing geriatric dental education, only publications from 2016 onwards were included. Full texts of the selected articles and those with unclear eligibility from titles or abstracts were further assessed for suitability. Manual searching of reference lists from included publications was also performed to identify additional relevant studies. Disagreements were resolved through discussion with a third researcher (CHC).

### 2.3. Data Extraction and Analysis

The two researchers extracted the data independently using a Microsoft Excel spreadsheet, with verification by a third researcher (CHC). Extracted information included: first author’s name, publication year, country, study type, participants, focus of study (barriers/facilitators/both), key barriers identified, key facilitators identified, strategies or interventions employed, and key findings. The two researchers independently conducted a thematic analysis by reviewing and familiarizing themselves with the extracted data and then coding meaningful segments related to barriers, facilitators, strategies, and interventions using an inductive approach manually in Microsoft Excel. Coding disagreements were discussed and resolved through consensus, with the third researcher (CHC) consulted when necessary. No formal assessment of coding reliability was performed. These codes were subsequently grouped into broader themes through an iterative process involving repeated comparison and refinement to ensure that they accurately reflected the data [15].

### 2.4. Critical Appraisal

In accordance with the PRISMA-ScR guidelines for scoping reviews, no formal quality assessment of the included studies was performed.

## 3. Results

### 3.1. Study Selection

**Figure 1:**
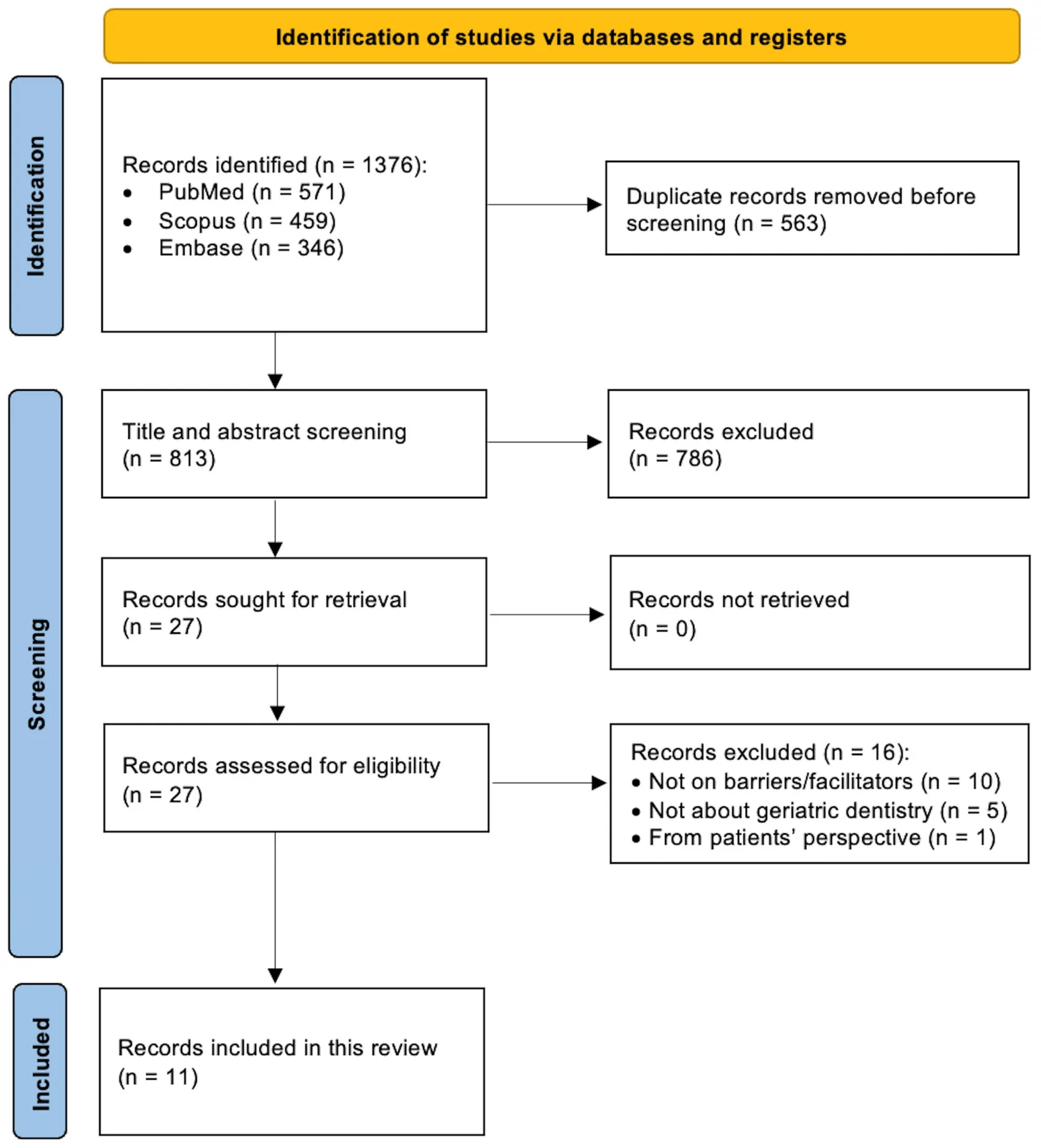
Flowchart of the literature search.

A total of 1376 publications were identified from three electronic databases: 571 from PubMed, 459 from Scopus, and 346 from Embase. After removing 563 duplicates, the titles and abstracts of 813 publications were screened according to the inclusion and exclusion criteria. Following this screening, 786 publications were excluded based on the exclusion criteria. The full texts of the remaining 27 publications were retrieved and assessed for eligibility. Sixteen publications were further excluded because they did not focus on barriers or facilitators, were not about geriatric dentistry education, or were from the patients’ perspective. Ultimately, eleven publications were included in this review for qualitative analysis. Figure 1 presents the flowchart of the literature search.

### 3.2. Study Characteristics and Key Findings

Among the eleven included studies, two were conducted in North America, five in Europe, two in Asia and two in Oceania. Seven studies employed a cross-sectional design, whereas four were prospective cohort studies. Eight studies collected qualitative data and four presented quantitative findings, with one of the eleven studies employing a mixed-methods approach. The included studies involved a total of 516 undergraduate dental students, 52 dental academics, and 8 stakeholders (representatives from older adult advocacy groups and directors of nursing in aged care facilities). Five studies focused on key barriers to implementing geriatric dental education, while all included studies investigated facilitators. Table 2 summarizes the characteristics and key findings of the included studies.

### 3.3. Themes of Barriers and Facilitators

Based on the included studies, three themes of barriers to implementing geriatric dental education were identified: structural and workforce-related barriers (resource constraints and a lack of equipped/trained staff), curricular and regulatory barriers (overcrowded curricula and a lack of curriculum guidelines), and interprofessional and attitudinal barriers (limited interprofessional collaboration and negative attitudes of students and teaching staff towards treating older patients).

**Figure 2:**
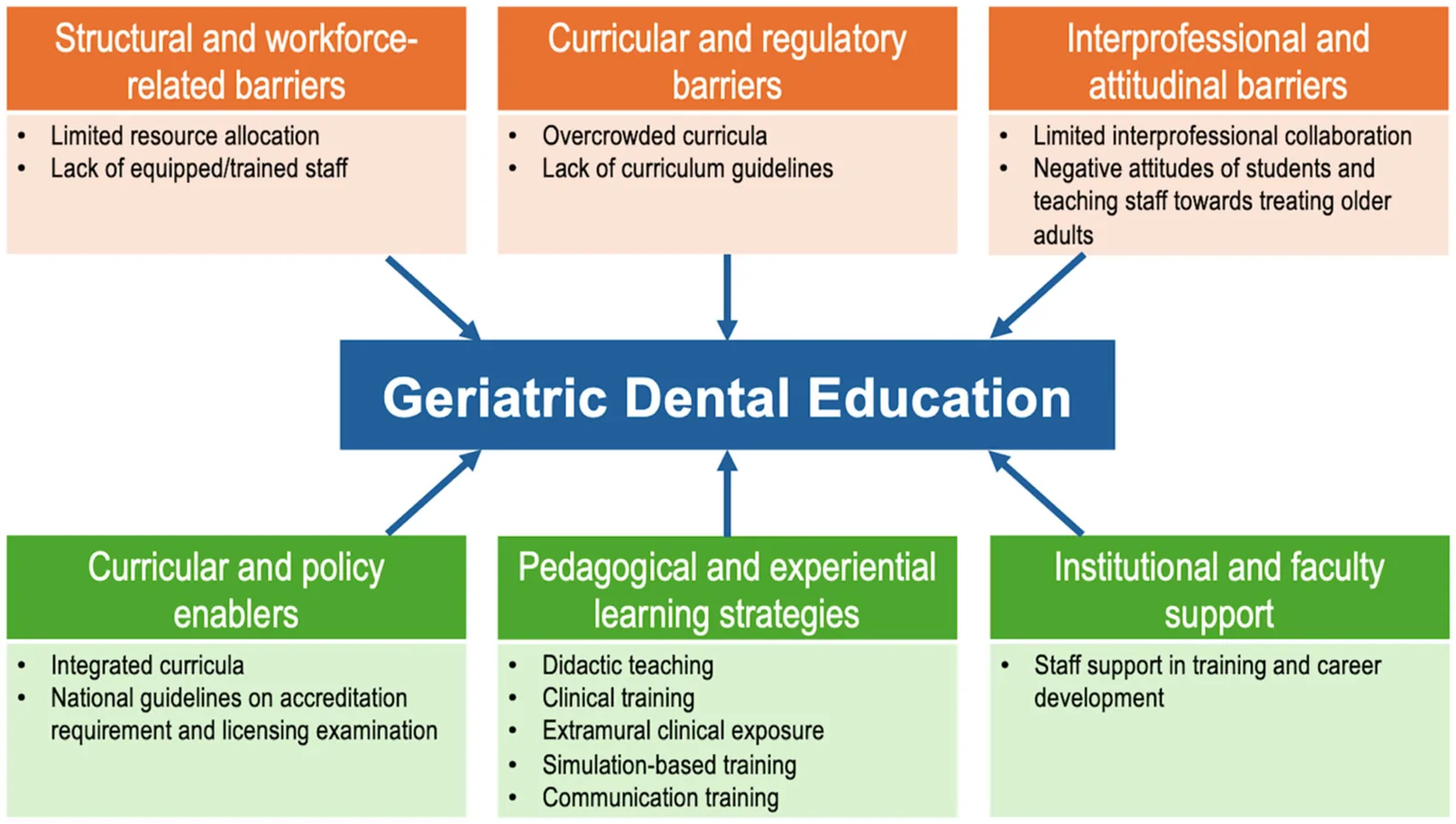
Barriers to and facilitators of undergraduate geriatric dental education.

Conversely, three themes of facilitators were recognized: pedagogical and experiential learning strategies (didactic teaching, clinical training, extramural clinical exposure, simulation-based training and communication courses); curricular and policy enablers (integrated curricula and national guidelines); and institutional and faculty support (staff support). Figure 2 presents the themes of barriers to and facilitators of undergraduate geriatric dental education identified via an inductive thematic analysis of the data extracted from the included studies.

### 3.4. Barriers to Undergraduate Geriatric Dental Education

#### 3.4.1. Structural and Workforce-Related Barriers

Limited resource allocation: Clinical training is resource-intensive, requiring a small-group teaching format, a specially trained supervisor, and facilities capable of accommodating vulnerable older patients. Many universities did not allocate sufficient funding to support hiring staff trained in geriatric dentistry or special care dentistry [20]. Moreover, budget constraints often limit faculty staff for supervision, as academic staff are burdened with administrative duties, further impeding the development of clinical training opportunities [21]. This lack of resources significantly impedes the integration of clinical components into undergraduate geriatric dental education [18].

Lack of equipped/trained staff: High-quality supervision is crucial for effective clinical learning, especially in geriatric dentistry, where understanding older patients’ unique characteristics and needs is vital for treatment planning and execution. A shortage of trained and motivated staff impairs the delivery of quality geriatric dental education [20,21]. Dental students reported that the quality of supervision significantly affected their clinical learning, with those supervised by skilled teaching staff gaining more clinical tips and knowledge beyond theoretical teaching. Dental students also expressed that trained supervisors were more prepared and willing to respond to their inquiries. Sadly, dental students often relied on “luck” to be assigned to skilled supervisors, limiting their clinical exposure [21]. Teachers also lacked confidence in training students to manage older patients with comorbidities, as they felt unprepared to employ a multidisciplinary approach [17]. The paucity of trained staff also hampers the opportunities for undergraduate students to gain some real-life clinical experience through placements in community centers, day care centers or elderly homes [23].

#### 3.4.2. Curricular and Regulatory Barriers

Overcrowded curricula: Geriatric dentistry is often dispersed across multiple disciplines, making it difficult to introduce as a dedicated standalone course within an already crowded curriculum. Adding a new course or module risks displacing or deleting existing content, which is problematic given the limited available time [23]. Furthermore, many dental schools are under pressure to meet accreditation requirements that constrain curriculum flexibility. Dental academics are worried that including an additional geriatric dental program or learning experience may interfere with their ability to fulfill these requirements within the designated timeframes [18]. They are also concerned that adding a separate course may overload the already crowded curriculum [17].

Lack of curriculum guidelines: In Australia, dental students reported significant variation in geriatric dental programs, particularly regarding the balance between didactic education and clinical training. Additionally, their learning depended largely on their allocation of clinical practice and the clinical skills of their supervisors. Without clear guidelines on the scope and depth of curriculum content, students feel uncertain about whether they are adequately prepared to deliver oral care to older adults in the future [21].

#### 3.4.3. Interprofessional and Attitudinal Barriers

Limited interprofessional collaboration: Given that older patients often present with multiple comorbidities and polypharmacy, interprofessional collaboration is essential for holistic oral care. Collaboration with medical doctors, pharmacists, social workers, physiotherapists, nurses, psychologists, and oncologists can enhance the overall management of older adults [18]. However, both students and teachers felt this collaboration was inadequate within undergraduate curricula. Barriers included a lack of awareness among other health professionals about the importance of oral health to overall health, concern over privacy, and logistical challenges within universities [18,23].

Negative attitudes of students and teaching staff towards treating older adults: Geriatric dentistry is often perceived as less appealing compared to other disciplines like prosthodontics. Dental students feel that treating frail older patients, primarily through a non-invasive and simple approach, may not significantly enhance their clinical skills [20]. Additionally, the financial incentives for treating older patients after graduation are viewed as unappealing, especially considering the extra time and effort required to manage older people with functional decline [23]. Since many dental schools lack a dedicated geriatric dentistry discipline, students also perceived limited job opportunities in this field. These factors collectively diminish students’ enthusiasm for geriatric dental learning. Because geriatric dentistry is often integrated into other disciplines rather than taught as a standalone specialty course, staff and students felt it was undervalued by educational institutions and national dental organizations [20]. This integration makes it difficult for educators to secure research funding or career promotion, thereby reducing their motivation for further training or staying in this field.

Ageism negatively influences both students’ and teachers’ attitudes toward providing oral care to older adults [27]. Some consider treating this population to be too time-consuming or stressful [17]. A study indicated that dental students tend to harbor prejudices, overestimating medical problems and disabilities such as dementia, dependency, depression, hearing loss, loneliness, or the inability to use technology among older patients [28]. Ageism is a sociological barrier affecting not only dental education but also nursing and medical training [29,30]. Changing these attitudes is essential to foster a more positive learning environment for geriatric care.

Dental students often find it challenging to build rapport with older patients, whose perspectives and backgrounds may differ significantly from theirs. Without a good connection, students felt more anxious and stressed, especially when performing complex or invasive procedures, as they perceived older patients to have less functional reserve [18]. This lack of rapport can hinder their interest in geriatric dental learning.

### 3.5. Facilitators of Undergraduate Geriatric Dental Education

#### 3.5.1. Pedagogical and Experiential Learning Strategies

Didactic teaching: Dental students agreed that didactic teaching provided essential foundational knowledge for delivering geriatric oral care. Key content areas include age-related changes, common medical and dental problems of older adults, treatment goals and planning for older adults, differences between normal aging and pathological conditions, techniques to enhance older patient comfort during dental treatment, common impairments and disabilities in older adults, and fostering empathy for the older population [18].

Clinical training: Hands-on clinical experience enhances students’ preparedness and skills in treating older adults. It also positively influences their attitudes towards geriatric care [20]. Dental academics emphasized that a lower student-to-supervisor ratio is crucial for effective clinical training [18]. Additionally, students have expressed that case discussions guided by trained staff in geriatric dentistry could enrich clinical exposure [18]. A cohort study found that students who received early clinical training perceived themselves as more competent in knowledge, communication, diagnosis and treatment planning in geriatric dentistry than those with later clinical exposure [26].

Extramural clinical exposure: Clinical experience outside university dental hospitals, such as in community centers or outreach programs, offers valuable real-life clinical exposure to dental students and increases their confidence in managing older patients [17,23]. Attard et al. [25] reported that 54% of dental students felt extramural clinical placements adequately prepared them to manage older patients, particularly those with diverse medical or physical conditions. Such experience helped students develop empathy and understand the broader needs of older adults [25]. However, some students found treating frail older adults challenging and reported feeling depressed, sad, and uncertain, especially when confronted with issues related to loneliness or illness [25]. Another study also reported that students perceived the nursing home experience as depressing [17].

To optimize extramural clinical experiences, students should start with low-risk interactions, such as oral health screenings or social visits in long-term care facilities or community centers [18]. Screening should be based on the older patients’ medical and physical conditions, and allocation should match the students’ level of clinical experience and competency to avoid negative experiences [18]. Supervisors should offer constructive feedback, and debriefing sessions should be conducted afterwards to support students’ emotions, with additional school counseling services provided if needed [17,25].

Simulation-based training: First encounters with real older patients can be stressful, especially for dental students unfamiliar with aging or disabilities [22]. Face-to-face simulation-based learning has been implemented as pre-clinical training in geriatric education in medicine. Professional actors as patients with different disabilities, like dementia, have been hired for verbal and nonverbal communication skill training. These simulation sessions can supplement the hardest topics, which are difficult to convey in didactic teaching [31].

Three included studies explored the effectiveness of simulation-based learning in geriatric dental education [16,22,24]. In the study by Germa et al., a serious game using a virtual clinical scenario and a patient with an avatar teacher was developed to enhance geriatric dental education in communication, patient assessment, and diagnosis and treatment planning. Students received real-time comments with positive/negative feedback icons or theoretical feedback as guidance during training. In this study, the majority of the students (more than two-thirds) felt more comfortable approaching older patients after training with the serious game [16]. In Patel et al.’s study, dental students wore an aging simulator to experience reduced self-care abilities and disabilities in vision, hearing, and physical movement experienced by older patients. Students reported having a better awareness of these four age-related disabilities and improved empathy towards older patients. Through the simulation, students also learned to modify their clinical practice to accommodate older patients with disabilities [22].

Communication training: Effective communication is vital, especially when caring for older patients with dementia or hearing loss [23]. Courses focused on enhancing communication and listening skills are considered essential for preparing dental students to serve the older population [18]. One included study found that direct engagement with community-dwelling older adults outside clinical settings allows students to practice communication without the pressure of treatment concerns, fostering more genuine interactions [19].

#### 3.5.2. Curricular and Policy Enablers

Integrated curricula: Considering the complex medical and physical conditions of older adults, a holistic, multidisciplinary approach is necessary [32]. An integrated dental curriculum that involves various disciplines can train dental students to manage older patients using a patient-centered approach rather than disease-specific treatment. Interprofessional education can further enable students to develop a team-based approach when caring for older patients with comorbidities or disabilities [17,23]. In Germany, the gerodontology training curriculum invited generalists and geriatricians to educate dental students about various medical geriatric conditions, including visual impairment, chronic obstructive pulmonary disease, tremor, hemiparesis, and kyphosis. This interprofessional education not only increased their preparedness in treating medically compromised older patients but also developed empathy for geriatric patients with physical disabilities [24].

National guidelines: Strong advocacy from national dental organizations and policymakers is essential for integrating geriatric dentistry into dental curricula [17]. National and international guidelines can mandate the inclusion of geriatric dental content in undergraduate programs [23]. For example, the Australian Dental Council has listed managing special care patients, including the elderly, as one of the competency requirements in the accreditation standards for dental schools [20]. Furthermore, incorporating geriatric dentistry into national licensing examinations can facilitate its integration into dental education [17].

#### 3.5.3. Institutional and Faculty Support Mechanisms

One of the institutional and faculty support mechanisms is support for staff development. Academics in India reported gaining geriatric dentistry knowledge and skills through continuing education programs and participation in national or international conferences. The Prosthodontic Society in India has facilitated training by sending groups to Japan, which has a well-established geriatric dental education program.

Academics also suggested that institutions should create career development awards and prioritize job openings for professionals trained in geriatric dentistry to ensure the quality of clinical supervision and reduce faculty workload. Expanding job opportunities in this field can also increase its attractiveness to students and help combat the perception that geriatric dentistry is undervalued within academic faculties [17].

## 4. Discussion

This scoping review maps current evidence on the barriers and facilitators influencing the implementation and development of undergraduate geriatric dental education. The findings indicate that geriatric dentistry is affected by multiple interacting factors at the educational, institutional, workforce, attitudinal, and policy levels. Although the importance of preparing future dentists for an aging population is increasingly recognized, undergraduate training remains inconsistent across countries and dental schools. This inconsistency is concerning because general dental practitioners are often the main providers of oral healthcare for older adults. Therefore, graduates should be equipped with appropriate knowledge, clinical skills, communication strategies, and professional attitudes to effectively manage older patients with complex medical, functional, cognitive, and social needs [10,11].

Resource constraints emerged as a major barrier in the included studies. Clinical training is vital in geriatric dental education, often requiring small-group supervision, trained clinical staff, accessible facilities, longer appointment times, and specialized equipment, particularly for older patients with mobility limitations or complex medical conditions. These requirements make geriatric clinical training resource-intensive. Several studies reported that limited funding, insufficient staffing, and inadequate infrastructure restricted opportunities for students to gain meaningful clinical experience with older adults [18,20,21]. Without adequate institutional investment, geriatric dentistry may remain largely theoretical, which is unlikely to sufficiently prepare students to provide safe and effective care for frail or medically compromised older patients.

Furthermore, the shortage of trained and motivated teaching staff limits the quality of geriatric dental education. High-quality supervision is essential because older patients often present with multimorbidity, polypharmacy, frailty, cognitive impairment, or physical disability. Students need guidance not only in dental procedures but also in diagnosis, treatment planning, communication, and interprofessional collaboration. Dental students reported that the quality of supervision significantly influenced their clinical learning; however, access to skilled supervisors varied [21]. Additionally, teachers expressed a lack of confidence in training students to manage medically compromised older patients [17]. These findings suggest that faculty development is central to curriculum improvement. Continuing education, clinical training, protected teaching time, academic career pathways, and institutional recognition of geriatric dentistry may help enhance teaching capacity and retain experienced staff [17].

Overcrowded curricula were another frequently reported challenge. Dental curricula are densely scheduled, and the addition of a separate geriatric dentistry course may be perceived as competing with existing disciplines [17,18,23]. In many dental schools, geriatric content is dispersed across disciplines such as prosthodontics, periodontology, special care dentistry, and community dentistry. This approach has potential advantages because oral care for older adults is inherently multidisciplinary. However, it can also lead to fragmented learning if the content is not well-coordinated. Students may encounter older patients in different clinical settings without receiving a coherent framework for understanding aging, frailty, functional decline, and medical complexity. Therefore, an integrated curriculum that is intentional, structured, and supported by clearly defined learning outcomes may be more effective for teaching patient-centered, interdisciplinary geriatric oral care.

The lack of standardized curriculum guidelines also contributes to variability in educational quality. Without clear national or institutional standards, dental schools may differ substantially in the amount, timing, and depth of geriatric dental education. Consequently, some students may graduate with substantial clinical exposure to older adults, while others may have very limited experience [21]. Implementing national guidelines, accreditation standards, and licensing requirements could help ensure that all students receive core training in geriatric dentistry. For example, the Australian Dental Council includes the management of special care patients, like older adults, within its competency requirements [20]. A similar policy could encourage dental schools to allocate appropriate curriculum time, staff, and resources to geriatric dentistry.

Interprofessional collaboration emerged as an important yet underdeveloped component of undergraduate geriatric dental education. Older adults often require multidisciplinary care involving dentists, physicians, nurses, social workers, physiotherapists, psychologists, and other health professionals [32,33]. Therefore, dental students should learn how to communicate and collaborate effectively within broader healthcare teams. However, the included studies reported limited interprofessional education in undergraduate curricula. Barriers included logistical difficulties, privacy concerns, institutional separation among disciplines, and limited awareness among health professionals of the importance of oral health [18,23]. Strengthening interprofessional education may better prepare students for collaborative practice in hospitals, care homes, community settings, and domiciliary care [11].

Negative attitudes towards older adults also hinder the development of geriatric dental education. Some students and teaching staff perceive geriatric dentistry as less appealing than other disciplines. Treating frail older patients may be viewed as time-consuming, emotionally demanding, and less financially rewarding [17,20,23]. Ageism can further diminish students’ interest and willingness to provide care for older adults [27,28]. These attitudes matter because they can influence students’ engagement during training and their future practice after graduation. Therefore, undergraduate geriatric dental education should not only address knowledge and clinical skills but also foster empathy and professional responsibility, challenging ageist perceptions. Strategies such as direct contact with older adults, reflective learning, communication training, and positive role modeling by teaching staff may help cultivate more positive attitudes.

These barriers persist primarily due to a lack of awareness about the importance of geriatric dentistry among educational institutions, academics and policymakers. Researchers should gather and present robust evidence on the growing aging population and their associated healthcare needs, emphasizing the critical role of geriatric dental care in public health. Additionally, they should demonstrate how improved oral health outcomes in older adults can reduce long-term healthcare expenses, thereby persuading policymakers to allocate greater resources and provide increased support for the development of geriatric dental education. Dental educators and curriculum developers should demonstrate how integrating geriatric dentistry into undergraduate curricula can enhance students’ competencies, improve patient outcomes and align with public health priorities to build broader awareness and advocacy.

Several facilitators were identified to enhance geriatric dental education. Didactic teaching remains necessary, as students need foundational knowledge on topics such as age-related changes, common oral and systemic conditions, polypharmacy, functional decline, and principles of geriatric care [18]. However, lectures alone are insufficient. Experiential learning is crucial for building confidence and clinical competence. Early clinical exposure to older patients can improve students’ self-perceived abilities in communication, diagnosis, and treatment planning [26]. Clinical training should be progressive, beginning with lower-risk activities and advancing to more complex care as students develop their skills.

Extramural placements are particularly valuable, offering students opportunities to engage with older adults in real-world settings, such as community centers, outreach programs, and long-term care facilities. These experiences help students understand the social, functional, and environmental factors influencing oral health in older populations. They also foster communication skills and empathy [17,23,25]. However, extramural learning must be carefully designed, as some students find experiences in nursing homes emotionally disturbing, especially when encountering issues like frailty, dependency, loneliness, or severe illness [17,25]. Patient selection should align with students’ clinical experience and competence and should be coupled with appropriate supervision, feedback, debriefing, and emotional support.

Simulation-based training is another promising facilitator. Tools such as aging suits, virtual patient scenarios, serious games, and simulated patients can help students understand sensory impairment, mobility limitations, cognitive decline, and communication barriers before treating real patients [16,22,24]. These approaches may reduce anxiety, enhance empathy, and provide opportunities for repeated practice in a safe environment. Simulation is especially useful for teaching communication skills and management strategies for disability-related challenges that are difficult to convey through traditional didactic methods [31,34]. Although simulation cannot replace real patient care, it can strengthen readiness and ensure consistent exposure to clinical situations that students might not encounter in clinical practice.

At the policy and institutional levels, strong leadership is essential. National dental organizations, accreditation bodies, and dental schools can promote geriatric dentistry by integrating it into competency frameworks, assessments, and licensing requirements [17,20,23]. Additionally, dental schools should invest in faculty development, strengthen community partnerships, improve access to clinical facilities, and support educational research. Creating academic career pathways and prioritizing the recruitment of staff trained in geriatric dentistry can enhance supervision quality and help address the perception that the field is undervalued [17].

The scope of barriers to and facilitators of implementation may differ between high-income and low-income countries. High-income countries such as the USA and Germany highlighted barriers and facilitators regarding the utilization of advanced technologies such as simulation or virtual reality in geriatric dental education, whereas lower–middle-income countries like India emphasized challenges such as a lack of trained staff and limited resource allocation [17,22,24].

This review also highlights significant gaps in the current evidence. Most included studies were qualitative or small-scale cohort studies. While they provide valuable insights, there is limited longitudinal evidence on whether specific educational strategies improve graduate competence, clinical behavior, or patient outcomes. Future research should evaluate integrated curricula, simulation training, interprofessional education, and extramural placements using robust study designs. Although this review incorporated perspectives from dental students and academics, insights from policymakers, institutional leaders, patients, and caregivers are also needed. Emerging technologies, like virtual reality, artificial intelligence, teledentistry, interprofessional simulation and digital education, offer scalable approaches for geriatric dental training, but their educational effectiveness warrants further investigation. Various learning models in medical education, such as competency-based education and Entrustable Professional Activities, can also be considered and evaluated for integration into geriatric dental education [35].

This review has certain limitations. While a comprehensive search was conducted across three electronic databases, only eleven studies were included, which limits the generalizability of the findings. Additionally, restricting the included publications to English-language articles published after 2016 may have introduced language and publication bias. Furthermore, the included studies exhibited substantial methodological heterogeneity, which warrants caution when interpreting the strength of the evidence.

In summary, undergraduate geriatric dental education is shaped by modifiable barriers and facilitators across multiple levels. Improving this field requires coordinated action rather than isolated curriculum changes. Dental schools need structured curricula, trained faculty staff, sufficient resources, experiential learning opportunities, and supportive policies. As populations age, preparing dental graduates to provide safe, compassionate, and effective care for older adults should be regarded as an essential component of undergraduate dental education.

## 5. Conclusions

This scoping review identifies modifiable educational, institutional, workforce, attitudinal, and policy factors that influence the implementation and development of undergraduate geriatric dental education. Key barriers include limited resources, shortages of trained staff, overcrowded curricula, unclear curriculum guidelines, weak interprofessional collaboration, and negative attitudes towards older adults. Conversely, important facilitators encompass structured didactic teaching, early clinical exposure, extramural placements, simulation-based learning, communication training, integrated curricula, faculty development, and national guidelines. Addressing these factors requires coordinated efforts among dental schools, professional bodies, accreditation agencies, and policymakers. Strengthening undergraduate geriatric dental education can better prepare graduates to provide safe, compassionate, and age-responsive oral healthcare. By consolidating current evidence, this study provides a foundation for future curriculum development and policy planning.

## Figures and Tables

**Table 1:** Eligibility criteria for scoping review of barriers and facilitators in undergraduate geriatric dental curricula.

	Inclusion Criteria	Exclusion Criteria
Population	Dental students or graduates Dental academics, educators, or curriculum developers in undergraduate programs Academic institutions involved in curriculum planning Policymakers, national dental organizations or stakeholders involved in shaping dental education	Academics, educators, curriculum developers or stakeholders involved in shaping postgraduate education, specialist training or continuing professional development Oral health professionals such as dental assistants, hygienists or therapists Patients
Concept	Focus on barriers and facilitators in delivering undergraduate geriatric dental education	Not focused on barriers or facilitators related to geriatric dental education
Context	Undergraduate educational settings, including dental schools, university clinical settings, or community-based academic programs with geriatric dentistry training	Settings outside undergraduate education, such as continuing professional development, postgraduate programs, or community outreach programs without an academic curriculum focus
Studies	Original research articles	Editorials, commentaries, reviews, conference proceedings, dissertations
Language	English	Non-English
Time	1 January 2016 to 31 May 2026	Before 2016

**Table 2:** Characteristics and key findings of the included studies.

First Author, Year, Country	Study Design, Participants	Key Barriers	Key Facilitators
Germa, 2025 [16], France	Cohort, qualitative 173 students	Not reported	Simulation-based learning
Shigli, 2025 [17], India	Cross-sectional, qualitative 28 academics	Negative attitudes Overcrowded curricula Lack of equipped/trained staff	Staff support National guidelines Integrated curricula Extramural clinical exposure
Brandt, 2024 [18], Canada	Cross-sectional, qualitative 10 students 9 academics	Negative attitudes Overcrowded curricula Limited resource allocation Limited interprofessional collaboration	Didactic teaching Clinical training Communication training Extramural clinical exposure
Gallagher, 2024 [19], United Kingdom	Cohort, qualitative 8 students	Not reported	Extramural clinical exposure
Nilsson, 2024 [20], Australia	Cross-sectional, qualitative 6 academics	Negative attitudes Lack of equipped/trained staff Limited resource allocation	Clinical training National guidelines
Nilsson, 2024b [21], Australia	Cross-sectional, qualitative 7 students 8 stakeholders	Lack of equipped/trained staff Limited resource allocation Lack of curriculum guidelines	Clinical training Integrated curricula
Patel, 2024 [22], United States	Cohort, mixed-methods 78 students	Not reported	Simulation-based learning
Prosser, 2022 [23], United Kingdom	Cross-sectional, qualitative 9 academics	Negative attitudes Overcrowded curricula Limited interprofessional collaboration	Extramural clinical exposure
Wighton, 2022 [24], Germany	Cohort, quantitative 44 students	Not reported	Simulation-based learning Integrated curricula
Attard, 2018 [25], Malta	Cross-sectional, quantitative 27 students	Not reported	Extramural clinical exposure
Patil, 2016 [26], India and Japan	Cross-sectional, quantitative 169 students	Not reported	Clinical training

## Data Availability

No new data were created or analyzed in this study. Data sharing is not applicable to this article.

## Supplementary Materials

The following supporting information can be downloaded at https://www.mdpi.com/article/10.3390/dj14090548/s1, Table S1: Preferred Reporting Items for Systematic Reviews and Meta-Analyses extension for Scoping Reviews (PRISMA-ScR) Checklist; Document S1: Search strategy.
